# Differential diagnosis between frontotemporal dementia and bipolar disorder, review and case report

**DOI:** 10.1192/j.eurpsy.2024.1324

**Published:** 2024-08-27

**Authors:** M. García Moreno, A. De Cos Milas, L. Beatobe Carreño, P. Del Sol Calderón, A. Izquierdo de la Puente

**Affiliations:** ^1^Psychiatry, Hospital Universitario Puerta de Hierro Majadahonda; ^2^Psychiatry, Hospital Universitario de Móstoles, Madrid, Spain

## Abstract

**Introduction:**

Dementia can present with psychiatric symptoms even before the cognitive impairment, which makes difficult to establish an adequate diagnosis. There have described symptoms of this type in vascular dementia, frontotemporal dementia, Alzheimer disease and Lewy bodies dementia. Frontotemporal dementia has a prevalence of 9-20% and it`s the third in frequency among degenerative dementia. It appears before the age of 65 years old and is more common in men. Two variants have been described, linguistic and behavioral. The behavioral one has usually an initial psychiatric presentation, with behavioral disorders, disinhibition and personality changes. Therefore it`s important to make an adequate differential diagnosis with late onset bipolar disorder.

**Objectives:**

To review about frontotemporal dementia and its differential diagnosis with late onset bipolar disorder.

**Methods:**

We carry out a literature review about frontotemporal dementia and its differential diagnosis with late onset bipolar disorder, accompanied by a clinical description of one patient with behavioral disturbance and language disorder.

**Results:**

A 59-year-old female was admitted to the short-term hospitalization unit from the emergency department due to behavior disorder. She had no relevant personal or familiar psychiatric history up to two years before when she received diagnosis of bipolar disorder. She presented behavioral disorganization, psychomotor restlessness, verbal aggressiveness, verbiage, insomnia and decreased intake. Psychopathological examination became difficult due to her language disorder since she presented an incoherent speech with paraphasias and loss of the common thread. Neurological study guided diagnosis to frontotemporal dementia even though they left the psychopharmacological treatment to our discretion. Olanzapine 5 mg twice a day was initiated, and behavioral improvement was observed. However, the patient maintained a significant functional impairment.

**Conclusions:**

Psychiatric presentation is frequent in dementia, even before cognitive failures which makes essential an exhaustive differential diagnosis. It`s important to consider the diagnosis of frontotemporal dementia in those patients who debut with behavioral disturbance in the 50s. Psychopharmacological treatment is only symptomatic so functional recovery should not be expected.

**Disclosure of Interest:**

None Declared

